# The apple 14-3-3 gene *MdGRF6* negatively regulates salt tolerance

**DOI:** 10.3389/fpls.2023.1161539

**Published:** 2023-04-03

**Authors:** Yuqing Zhu, Wei Kuang, Jun Leng, Xue Wang, Linlin Qiu, Xiangyue Kong, Yongzhang Wang, Qiang Zhao

**Affiliations:** ^1^ College of Horticulture, Qingdao Agricultural University, Qingdao, Shandong, China; ^2^ Engineering Laboratory of Genetic Improvement of Horticultural Crops of Shandong Province, Qingdao Agricultural University, Qingdao, Shandong, China; ^3^ Laboratory of Quality & Safety Risk Assessment for Fruit (Qingdao), Ministry of Agriculture and Rural Affairs, Qingdao Agricultural University, Qingdao, Shandong, China; ^4^ Qingdao Key Laboratory of Modern Agriculture Quality and Safety Engineering, Qingdao Agricultural University, Qingdao, Shandong, China

**Keywords:** apple, 14-3-3 proteins, *MdGRF6*, salt stress, negative regulation

## Abstract

The 14-3-3 (GRF, general regulatory factor) regulatory proteins are highly conserved and are widely distributed throughout the eukaryotes. They are involved in the growth and development of organisms *via* target protein interactions. Although many plant 14-3-3 proteins were identified in response to stresses, little is known about their involvement in salt tolerance in apples. In our study, nineteen apple 14-3-3 proteins were cloned and identified. The transcript levels of *Md14-3-3* genes were either up or down-regulated in response to salinity treatments. Specifically, the transcript level of *MdGRF6* (a member of the *Md14-3-3* genes family) decreased due to salt stress treatment. The phenotypes of transgenic tobacco lines and wild-type (WT) did not affect plant growth under normal conditions. However, the germination rate and salt tolerance of transgenic tobacco was lower compared to the WT. Transgenic tobacco demonstrated decreased salt tolerance. The transgenic apple calli overexpressing *MdGRF6* exhibited greater sensitivity to salt stress compared to the WT plants, whereas the *MdGRF6-RNAi* transgenic apple calli improved salt stress tolerance. Moreover, the salt stress-related genes (*MdSOS2*, *MdSOS3*, *MdNHX1*, *MdATK2/3*, *MdCBL-1*, *MdMYB46*, *MdWRKY30*, and *MdHB-7*) were more strongly down-regulated in *MdGRF6-OE* transgenic apple calli lines than in the WT when subjected to salt stress treatment. Taken together, these results provide new insights into the roles of 14-3-3 protein MdGRF6 in modulating salt responses in plants.

## 1 Introduction

Plants are often affected by environmental stresses such as high salinity, drought, and extreme temperatures, which can adversely inhibit their growth and development (Golldack et al., 2014). High salinity is a severe abiotic stressor that inhibits plant development and productivity (Yoshida et al., 2014). Many genes are involved in salt stress tolerance, including antioxidant protective enzymes, signal transduction, and transcription factors (TFs) (Pardo et al., 1998; Mao et al., 2017; Jiroutova et al., 2021). Among these, 14-3-3 proteins primarily act as molecular chaperones and are extensively involved in abiotic and biotic stress responses *via* regulation of the conformation, activity, stability, and subcellular localization of target proteins (Campo et al., 2012).

The 14-3-3 proteins are a family of highly conserved proteins found in all eukaryotes, and can be classified into two categories: the non-ϵ (non-epsilon) and ϵ (epsilon) types (Brennan et al., 2013; Cao et al., 2016). The target proteins of the 14-3-3 family are phosphorylated at certain sites, causing a conformational shift that allows the family to exist as homo or heterodimers (Yasuda et al., 2014). In plants, protein interactions allow the 14-3-3 protein to attach to the plasma membrane H^+^-ATP (Jahn et al., 1997). This interaction, which mediates the ATP-driven transport of H^+^ across membranes, is involved in the active transport to manage both the osmotic and ionic stresses under high-salinity conditions (Wang et al., 2021). The 14-3-3 protein characteristics allow for the control of a variety of environmental signaling pathways, including those related to drought, excessive salinity, and extreme temperatures (Li et al., 2013; He et al., 2015; Tian et al., 2015).

In recent years, a growing number of studies have investigated the molecular functions of the 14-3-3 proteins in plants. To date, 13, 8, 12, 18, 5, 6, and 11 14-3-3 proteins have been identified in *Arabidopsis* (*Arabidopsis thaliana*), rice (*Oryza sativa*), tomato (*Solanum lycopersicum*), soybean (*Glycine max*), barley (*Hordeum vulgare*), cotton (*Gossypium hirsutum*), and grape (*Vitis vinifera L*.), respectively (Xu and Shi, 2006; Schoonheim et al., 2007; Yao et al., 2007; Zhang et al., 2010; Li and Dhaubhadel, 2011). In tobacco, 14-3-3 proteins interact with RSG and CDPK1 to form a heterodimeric trimer, which is used to control the gibberellin pathway (Ito et al., 2014). In tobacco, A 14-3-3 protein is involved in biological stress response *via* the interaction with the plasma membrane oxidase (NtrbohD16) to regulate ROS production in plants (Elmayan et al., 2007). In response to heat, cold, and salt stresses, rice exhibits significant variations in *OsGRF* expression (Yao et al., 2007). The 14-3-3λ and 14-3-3κ proteins in *Arabidopsis* participate in response to salt stress by regulating SOS2 activity in the SOS (salt overly sensitive) pathway (Zhou et al., 2014). 14-3-3 proteins are involved in the browning pathway of potato tubers by regulating the antioxidant enzyme activity (Łukaszewicz et al., 2002). Additionally, 14-3-3 proteins demonstrate considerable up- or down-regulation in *Vitis vinifera L.* under cold and heat stresses, suggesting their possible function in the regulation of abiotic stress response (Cheng et al., 2018).

Plants cope with salt stress through complex physiological responses and molecular regulatory mechanisms. However, the precise role of apple 14-3-3 proteins in regulating salt stress remains largely unclear. Therefore, it is very valuable to clarify the function of 14-3-3 protein under salt stress. To identify the function of *Md14-3-3s*, a total of nineteen 14-3-3 genes in apples were analyzed in this study. Functional characterization demonstrated that *MdGRF6* negatively regulates salt tolerance. Therefore, this study provides the basis for future research examining the molecular processes of *MdGRF6* in controlling the salt stress response in apples.

## 2 Materials and methods

### 2.1 Plant materials and treatments

The apple (*Malus×domestica*) seedlings were cultured in MS medium with 1 mg/L 6-BA, 0.1 mg/L NAA, and 0.1 mg/L GA3 at 23 ± 1°C and 16 h light/8 h dark photoperiod. The apple calli were cultured for 20 days in the dark at room temperature (24°C) on MS medium with 3 mg/L 2, 4-D, and 0.4 mg/L 6-BA. Tobacco seeds were surface-sterilized with 2% sodium hypochlorite (NaClO) for 10 min followed by 75% ethanol wash for 1 min. Then the seeds were washed with sterile water five times and cultured on MS medium with 0.8% agar and 2.5% sucrose at 23°C under 16 h light/8 h dark photoperiod.

For tissue expression analysis, the samples (roots, stems, leaves, fruits, and seeds) were collected from ten five-year-old apple trees grown in the experimental field of the horticulture orchard of the Qingdao Agricultural University (Shandong Province, China) in October 2022. For gene expression, the apple seedlings were treated with 100 mmol/L NaCl and harvested at 0, 1, 3, 6, and 12 h after treatment. Following the collection of each sample, the seedlings were immediately flash-frozen in liquid nitrogen and stored at -80°C until future use.

### 2.2 RT-qPCR analysis

Total RNA was extracted using the RNA Plant Plus Reagent (Tiangen, Beijing, China) and the first-strand cDNA was synthesized using the PrimeScript First Strand cDNA Synthesis Kit (Takara, Dalian, China). The ChamQ SYBR Mixture (Vazyme) was used for RT-qPCR reactions using a LightCycler 480 II RT-qPCR system. The cycle threshold (Ct) 2^−ΔΔCT^ approach was used to analyze relative gene expression (Livak and Schmittgen, 2001). *MdACTIN* (apple) and *NtACTIN* (tobacco) were used as internal controls. Each RT-qPCR sample was done at least in triplicate. The primers are listed in **Supplemental Table S2**.

### 2.3 Bioinformatic analysis

Sequences of the 14-3-3 protein from apple, rice, and *Arabidopsis* were aligned using ClustalW in MAGEX. Neighbor-joining was used to construct the molecular phylogenetic trees with 1,000 reiterations. iTOL (https://itol.embl.de/itol.cgi) was used to annotate the 14-3-3 protein sequence information. TBtools was used to identify the chromosomal locations of *Md14-3-3* genes, using the apple genome annotation file (gene_models_20170612.gff3.gz) (Chen et al., 2020). Plant-CARE (http://bioinformatics.psb.ugent.be/webtools/plantcare/html/) was then used to analyze the *cis*-elements in the promoters of *Md14-3-3* genes (2,000-bp fragment at the upstream of the start codon) (Wang et al., 2014). Protein sequences of the *Md14-3-3* genes family were submitted to MEME (https://meme-suite.org/meme/doc/meme.html) for conserved motif analysis (Tripathi et al., 2016).

### 2.4 Plasmid construction and genetic transformation

The full-length of *MdGRF6* was cloned into the overexpression vector *pRI101-EGFP* to obtain the *35S::MdGRF6-EGFP* recombinant plasmids. The *MdGRF6-RNAi* were constructed using the RNAi vector. To generate the *P_MdGRF6_::GUS* reporter construct, ~2 kb *MdGRF6* promoter fragments were cloned from apple genomic DNA and inserted into the *pCAMBIA1391::GUS* vector containing the GUS reporter gene. The primers in this study are listed in **Supplemental Table S2**.

The recombinant vectors *35S::MdGRF6-EGFP*, *MdGRF6-RNAi*, and *35S::EGFP* were introduced into *Agrobacterium rhizogenes* strain EHA105. The transgenic apple calli or tobacco plants were generated by *Agrobacturium*-mediated transformation method as described previously (Gallois and Marinho, 1995; An et al., 2017). The calli were subcultured on the MS medium containing 30 mg/L kanamycin, and the rooted transgenic tobacco plants were planted into the soil.

### 2.5 Subcellular localization analysis of *MdGRF6*

For subcellular localization, the *35S::MdGRF6-EGFP* or *35S::EGFP* vector was transformed into an *Agrobacterium* EHA105 strain and used for transient expression in *Nicotiana benthamiana* leaves. The fluorescence was then observed after 2-3 days using a laser confocal microscope.

### 2.6 The histochemical GUS staining analysis

The 30-day *P_MdGRF6_::GUS* transgenic tobacco was used for tissue expression analysis. Young leaf, stem, and root tissues were visualized using the GUS Staining Kit (Solarbio, Beijing, China).

### 2.7 Salt stress treatment

The WT and transgenic apple calli were treated with 100 mM NaCl or 150 mM NaCl for 15 days. Following treatment, the apple calli were collected and the fresh weight was measured. Relative conductivity was determined according to Yang et al. method (Yang et al., 2022), using a DDSJ-318 conductometer (Yidian Scientific Instrument Co., Ltd., Shanghai, China). The thiobarbituric acid-based method was used to determine the malondialdehyde (MDA) content and the absorbance value of the reaction solution was determined using a spectrophotometer at 600, 532, and 450nm (Yoke Istrument Co., Ltd., Shanghai, China).

WT and transgenic tobacco seeds were sterilized and sown onto MS, MS + 50 mmol/L NaCl, or MS + 100 mmol/L NaCl plates to detect the germination rates. Following seed germination, consistently growing transgenic and WT tobacco were transferred to MS medium with 50 mM NaCl or 100 mM NaCl for 10 days. After salt treatment, the fresh weight, root length, chlorophyll, and MDA content were measured. As previously described, the MDA content was measured using the thiobarbituric acid method. Chlorophyll was extracted with 95% ethanol, and the supernatant was used to determine the absorbance values at 665 and 649 nm (Yoke Instrument Co., Ltd., Shanghai, China).

Uniformly-developed seedlings of transgenic and WT tobacco plants were treated with 350 mM NaCl for four weeks. Water was used as the control. After four weeks, the chlorophyll content and relative conductivity were determined as above described. Diaminobenzidine (DAB) and p-nitroblue tetrazolium chloride (NBT) histochemical staining were used to determine H_2_O_2_ and ${\text{O}}_{2}^{\;\cdot -}$ contents in tobacco leaves, respectively. The activities of enzymes SOD and POD were measured using the SOD and POD assay kits (Solarbio, Beijing, China), respectively. The above experiments were replicated three times.

## 3 Results

### 3.1 Identification and phylogenetic tree analysis of *MdGRF* genes

**Figure 1:**
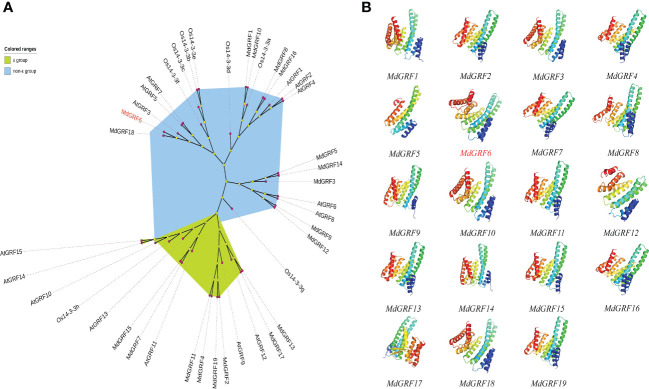
Phylogenetic tree and protein structures of Md14-3-3s. **(A)** The NJ phylogenetic tree (1,000 bootstrap replicates) was generated with 14-3-3 proteins from apples, rice, and *Arabidopsis*. **(B)** Phyre2 in ‘Normal’ mode and Pymol were used to generate all 3D models of MdGRFs. Images were colored by rainbow N → C terminus. All models were based on template d1o9da or c3e6yB.

To identify the 14-3-3 genes in the apple genome, the BLASTp and the Pfam tool were used. A total of twenty 14-3-3 genes were identified in the apple genome based on their homology to the 14-3-3 protein sequences in *Arabidopsis* from the TAIR database (http://www.arabidopsis.org). However, only nineteen genes were cloned and labeled as *MdGRF1*-*MdGRF19* based on their chromosomal location (**Table S1**). The phylogenetic tree demonstrated that the Md14-3-3 proteins were divided into two groups (ϵ and non-ϵ group) (**Figure 1A**). Following the protein 3D models, the 14-3-3 secondary structures were classified into three divisions. The middle position domain structure was similar, while the differences were the N-terminal and C-terminal structures (**Figure 1B**).

### 3.2 Analysis of *cis*-acting elements in the promoters of *Md14-3-3* genes

**Figure 2:**
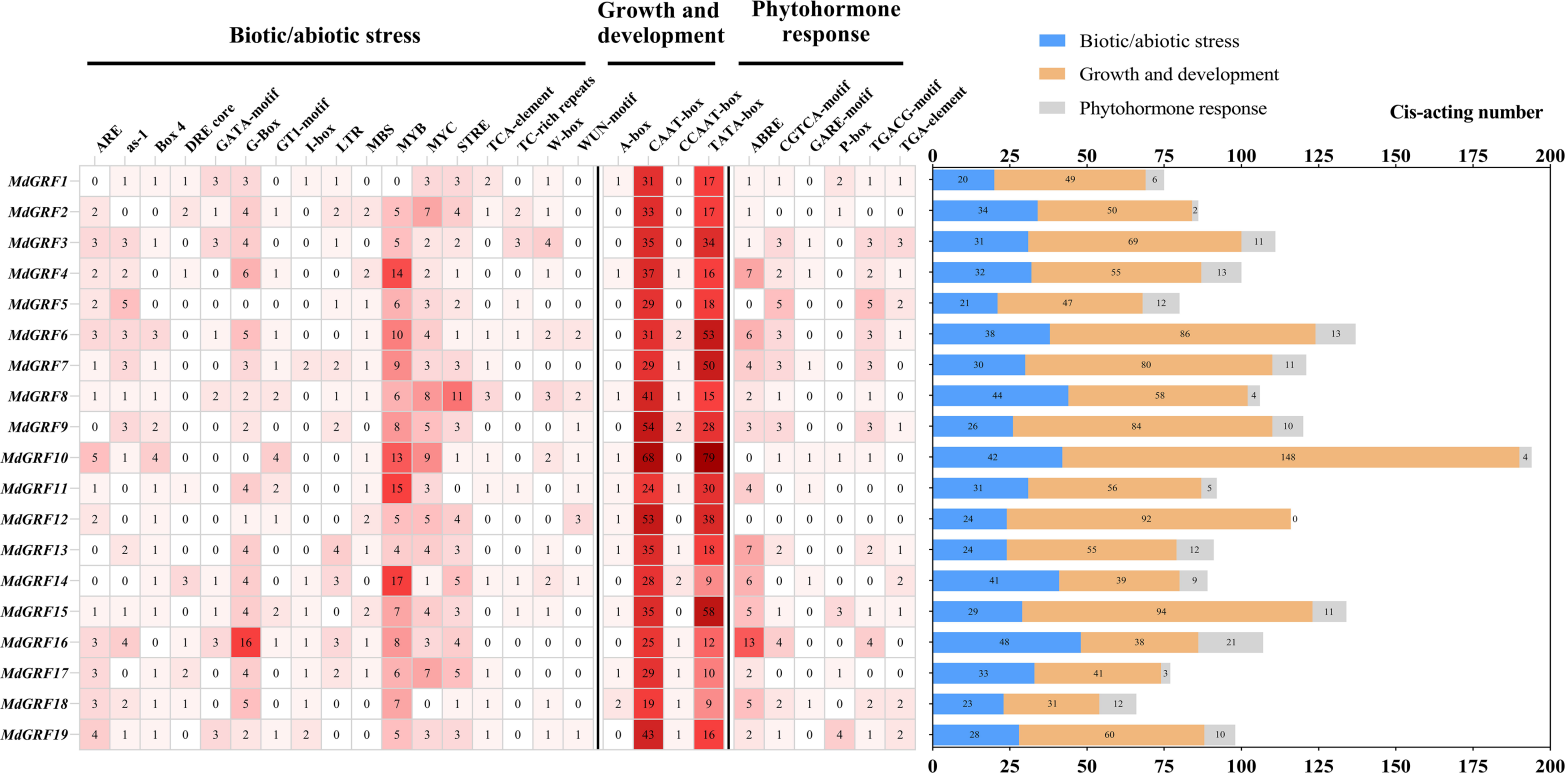
Promoter analysis of *Md14-3-3s*. The amount of various *cis*-acting elements are presented in the grid by the different numbers and color tones. The sum of the *cis*-acting elements in the three classes of each gene is displayed in the histogram.

The *cis*-acting elements in the promoter regions of *Md14-3-3* genes were examined using the PlantCARE database (**Figure 2**). Based on their functions, the *cis*-acting elements were divided into three groups: biotic/abiotic stress, growth and development, and phytohormone response. The *cis*-acting elements of plant biotic/abiotic stress, including salicylic acid (TCA-element: CCATCTTTTT), defense and stress (TC-rich repeats: GTTTTCTTAC), low temperature (LTR: CCGAAA), MYB-binding site involving in drought-inducibility (MBS: CAACTG), were identified in the promoters of the *Md14-3-3* genes. A large number of core *cis*-acting elements (such as TATA box, CAAT box, and others) were also identified in the *Md14-3-3s* promoter regions. In addition, multiple elements responding to phytohormones were identified. The promoters of *Md14-3-3s* also contained *cis*-acting elements responding to TGA-element (AACGAC), ABRE (ACGTG), GARE (TCTGTTG), and CGTCA that involved in auxin (IAA), abscisic acid (ABA), gibberellin (GA), and methyl jasmonate (MeJA) response.

### 3.3 Chromosome localization, gene structure, and conserved *Md14-3-3* motifs analysis

**Figure 3:**
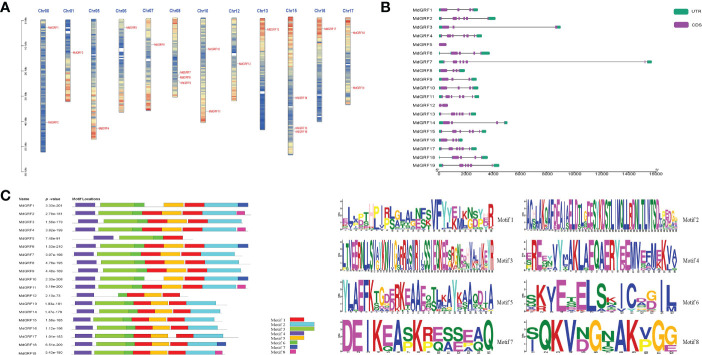
Characterization and chromosomal localization of the *Md14-3-3* genes. **(A)** Chromosomal localization of 14-3-3 genes in apple. **(B)** Structural analysis of the *Md14-3-3* genes. Different blocks represent UTRs (green), CDs (purple), and introns (black lines). **(C)** The motif details of the identified conserved domains of the Md14-3-3 proteins. Each motif is represented by a different colored box.

The *Md14-3-3* genes were identified on twelve apple chromosomes (Chr): Chr00, 01, 05, 06, 07, 08, 10, 12, 13, 15, 16, and 17 (**Figure 3A**). The intron/exon analysis showed that the *Md14-3-3* genes contained from 0 to 6 introns (**Figure 3B**). Among these, *MdGRF4*, *7*, *11*, *13*, *15*, *17*, and *19* contained the most introns (6 introns), while *MdGRF5* had none. Additionally, the phylogenetic tree demonstrated that *MdGRF6* and *18*, which were both situated on the same branch, had similar intron/exon distribution (**Figure 3B**). Eight motifs in the Md14-3-3 proteins were predicted using the Multiple Em for Motif Elicitation (MEME) program. Most of the conserved motifs were similar in distribution (**Figure 3C**). However, these conserved motif differences may be responsible for different gene functions.

### 3.4 Expression patterns of *MdGRF6* and subcellular localization

**Figure 4:**
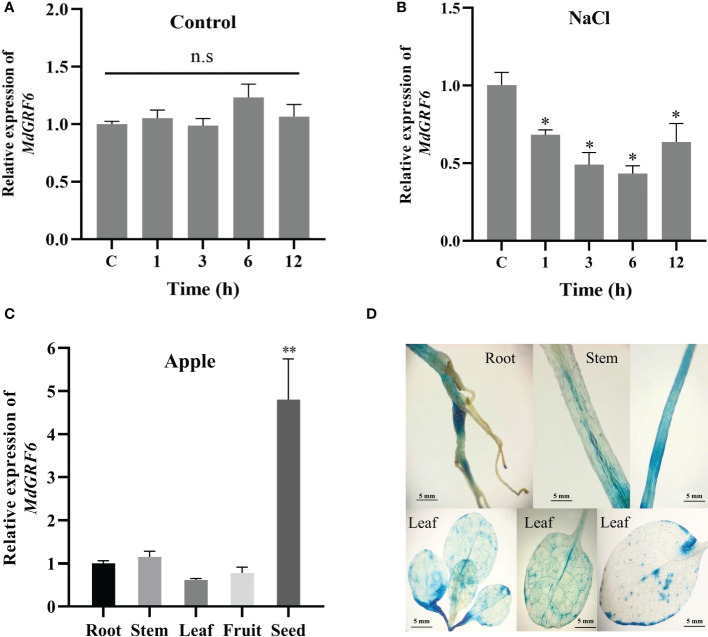
Expression patterns of *Md14-3-3* genes. **(A, B)** RT-qPCR analysis of *MdGRF6* expression in apple seedlings treated without or with 100 mM NaCl for the indicated time. **(C)** Expression analysis of *MdGRF6* in different apple tissues. **(D)** *P_MdGRF6_::GUS* expression pattern in transgenic tobacco in different tissues. Data are presented as means ± SD (n = 3). Asterisks indicate significant differences LSD test, **P < 0.01; *P < 0.05; ns, no significant difference.

Previous studies have shown that 14-3-3 genes play an important role in plant response to salt stress (Zhou et al., 2014). Here, the transcript levels of the *MdGRFs* family members were up or down-regulated in response to salinity treatments (**Figure S1**). One of the members, *MdGRF6* was chosen for further analysis. *MdGRF6* was down-regulated under salt treatment, with a 0.42-fold down-regulation at the sixth hour. However, there was no significant change in the control group (**Figures 4A, B**). Next, gene expression in apple roots, stems, leaves, fruits, and seeds were analyzed. The results demonstrated that *MdGRF6* was highly expressed in seeds, with the lowest expression in leaves (**Figure 4C**). To confirm *MdGRF6* these results, the *P_MdGRF6_::GUS* transgenic tobacco plants were then developed. GUS signals were detected in the roots, stems, and leaves of the transgenic tobacco seedlings (**Figure 4D**).

In addition, the constructed expression vectors *35S::MdGRF6-EGFP* or *35S::EGFP* were injected into *Nicotiana benthamiana* leaves to examine the subcellular localization of MdGRF6. As a result, we found that MdGRF6 was localized in the cytoplasm and cell membrane (**Figure S2**).

### 3.5 Overexpression of *MdGRF6* increased sensitivity to salt stress in transgenic tobacco

**Figure 5:**
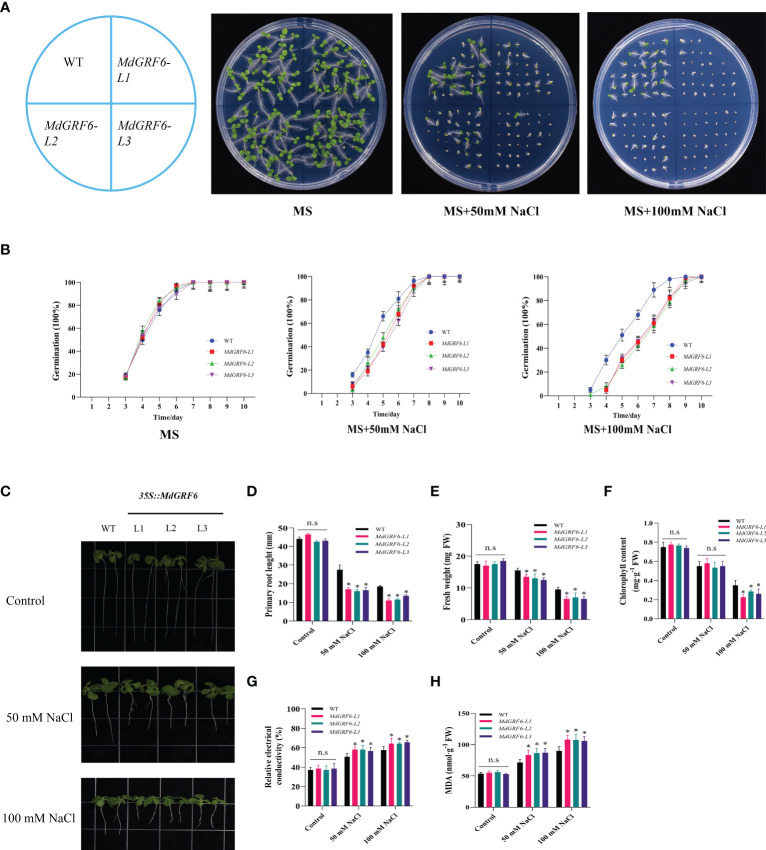
Salt response of *35S::MdGRF6* transgenic tobacco plants during germination. **(A)** Effects of salt treatment on the germination of WT and *35S::MdGRF6* transgenic tobacco lines. Germination was assessed at the indicated time. **(B)** The WT and *35S::MdGRF6* transgenic tobacco lines were grown on MS medium supplemented with 0, 50, or 100 mM NaCl. The seeds germinated after 10 days. Representative seedlings are presented in the images. **(C)** The seedlings were grown vertically for 4 days on MS medium and then moved to the medium containing different doses of 50 or 100 mM NaCl for an additional 10 days in a vertical position. **(D)** Root elongation of WT and *35S::MdGRF6* transgenic tobacco seedlings in response to salt stress. **(E–H)** Fresh weight **(E)**, chlorophyll content **(F)**, relative electrical conductivity **(G)**, and MDA content **(H)** in WT and *35S::MdGRF6* transgenic tobacco seedlings under control or salt stress conditions. Error bars indicate the means ± SD (n = 3). The asterisks indicate significant differences (LSD test, *P < 0.05; ns, no significant difference).

To characterize the function of *MdGRF6* under salt stress, three independent transgenic tobacco lines (#1, #2, and #3) were selected for further analyses using RT-qPCR and western blotting (**Figure S3**). The germination rates of the WT and *MdGRF6-OE* lines on MS medium with or without NaCl were first determined. The germination rates were comparable on MS-only media (**Figures 5A, B**). However, supplementation with 50mM or 100mM NaCl significantly reduced the germination rates of all lines, but had the most significant effects in the transgenic plants (**Figures 5A, B**). Furthermore, the roots were significantly shorter in the *MdGRF6-OE* transgenic plants compared to WT (**Figures 5C, D**). Under salt stress, *MdGRF6-OE* transgenic plants exhibited lower fresh weights and chlorophyll content compared to WT (**Figures 5E, F**), and exhibited significantly higher electrolyte leakage and MDA content compared to WT (**Figures 5G, H**).

**Figure 6:**
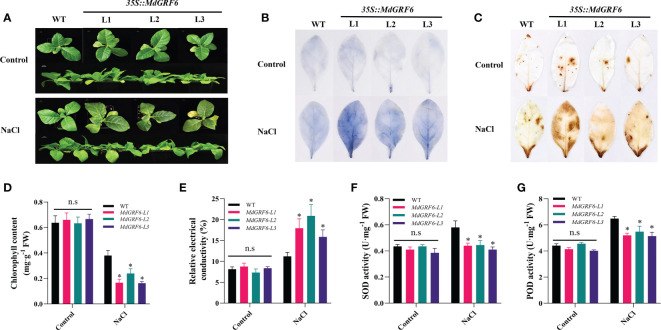
Effects of salt on the growth of germinated WT and *35S::MdGRF6* transgenic tobacco plants. **(A)** The phenotype of WT and three transgenic tobacco lines under salinity stress conditions. **(B, C)** Following salt stress treatments, leaves from *MdGRF6* transgenic lines and WT were stained for H_2_O_2_ using DAB staining and for ${\text{O}}_{2}^{\;\cdot -}$ using NBT staining. Statistical analysis of **(D)** chlorophyll content, **(E)** electrical conductivity, **(F)** SOD activity, and **(G)** POD activity in WT and transgenic lines after salt treatments. Error bars indicate the means ± SD (n = 3). The asterisks indicate significant differences (LSD test, *P < 0.05; ns, no significant difference).

In addition, 30-day-old soil-grown seedlings of WT and transgenic plants were supplied with water or 350 mM NaCl for four weeks. The results of observed phenotypes were consistent with result of germination rates (**Figure 6A**). ROS levels may increase in response to abiotic stress, and their accumulation is harmful to plant cells (Sun et al., 2010). Under salt treatment, DAB and NBT histochemical staining of transgenic leaves were more intense than in WT than *MdGRF6-OE* plants, and *MdGRF6* transgenic lines accumulated more H_2_O_2_ and ${\text{O}}_{2}^{\;\cdot -}$ contents compared to WT plants (**Figures 6B, C**). Consistent with this data, the activities of important antioxidant enzymes (POD and SOD) displayed lower in *MdGRF6-OE* plants compared to WT (**Figures 6F, G**). Furthermore, the increased electrical conductivity and decreased chlorophyll content further confirmed that *MdGRF6* overexpression reduced salt tolerance (**Figures 6D, E**). According to the above results, the ectopic expression of *MdGRF6* decreased tolerance to salt stress by impeding the activity of several antioxidant enzymes and causing H_2_O_2_ and ${\text{O}}_{2}^{\;\cdot -}$ accumulation.

### 3.6 *MdGRF6* negatively regulates salt stress tolerance in transgenic calli

**Figure 7:**
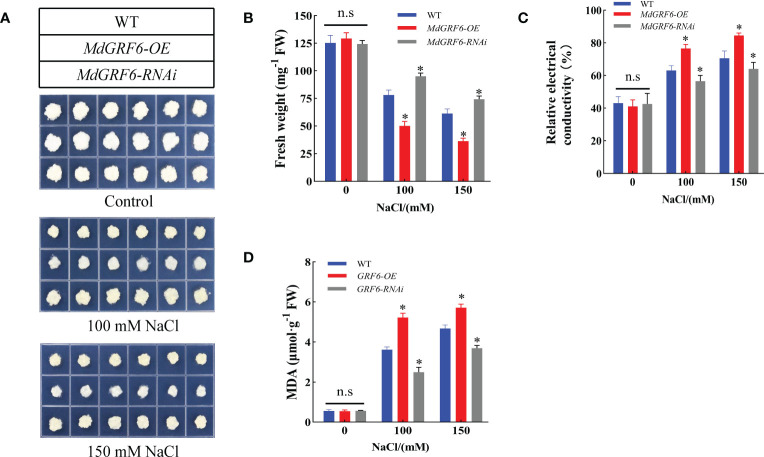
*MdGRF6* enhanced sensitivity to salt stress in apple. **(A)** The phenotypes of transgenic and WT apple calli in response to NaCl treatment. The apple calli were placed on the MS medium containing 100 or 150 mM NaCl for 15 days. **(B-D)** Fresh weight, electrical conductivity, and MDA content of the WT and transgenic apple calli after 15 days under salt treatment. Error bars indicate the means ± SD (n = 3). The asterisks indicate significant differences (LSD test, *P < 0.05; ns, no significant difference).

To further investigate *MdGRF6* function under salt stress, the *MdGRF6-OE* and *MdGRF6-RNAi* transgenic apple calli were obtained. RT-qPCR demonstrated that *MdGRF6-OE* and *MdGRF6-RNAi* transgenic calli generated significantly higher or lower expression levels compared to WT, respectively (**Figure S4**). Then, the 15-day-old WT, *MdGRF6-OE*, and *MdGRF6-RNAi* transgenic calli were placed on MS medium containing 100 mM or 150 mM NaCl. The results demonstrate that *MdGRF6-OE* transgenic calli grew significantly slower than WT, and *MdGRF6-RNAi* transgenic calli grew much stronger than WT (**Figure 7A**). In agreement with the observed phenotype, *MdGRF6-OE* calli exhibited higher MDA level, lower fresh weight, and higher electrical conductivity compared to the WT, whereas *MdGRF6-RNAi* transgenic calli reduced MDA content, electrical conductivity, and had higher fresh weight under salt stress (**Figures 7B–D**). Overall, the results suggest that *MdGRF6* overexpression improved sensitivity to salt in transgenic apple calli.

### 3.7 Overexpression of *MdGRF6* downregulates salt stress-related gene expression

**Figure 8:**
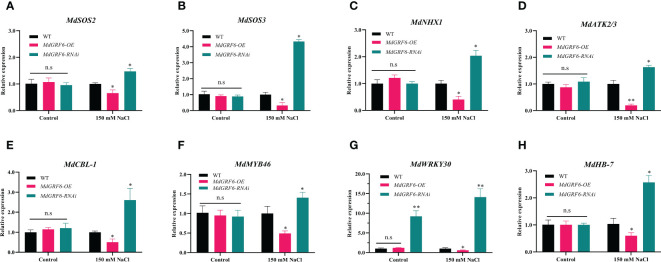
*MdGRF6* affected the expression of salt stress-related genes in response to salt stress. **(A–H)** The relative expression of *MdSOS2* **(A)**, *MdSOS3* **(B)**, *MdNHX1* **(C)**, *MdATK2/3* **(D)** *MdCBL-1* **(E)**, *MdMYB46* **(F)**, *MdWRKY30* **(G)**, and *MdHB-7* **(H)** in WT, *MdGRF6-OE* and *MdGRF6-RNAi* transgenic calli under control and salt treatments. Error bars indicate the means ± SD (n = 3). The asterisks indicate significant differences LSD test, **P < 0.01; *P < 0.05; ns, no significant difference.

To investigate the molecular mechanisms of *MdGRF6*-mediated salt stress tolerance, RT-qPCR was used to assess the expression levels of salt stress-related genes (*MdSOS2*, *MdSOS3*, *MdNHX1*, *MdATK2/3*, *MdCBL-1*, *MdMYB46*, *MdWRKY30*, and *MdHB-7*). Under normal conditions, the transcript abundance did not differ between the *MdGRF6-OE* transgenic calli and WT. However, the expression of these genes was considerably lower in *MdGRF6-OE* transgenic calli, and was higher in *MdGRF6-RNAi* transgenic calli compared to WT calli under salt stress (**Figures 8A–H**). The above results suggested that *MdGRF6* may negatively regulate these genes under salt stress.

## 4 Discussion

Salinization is one of the most increasingly severe environmental and ecological issues that threaten the limited soil resources on which humans depend and poses a significant constraint on the sustainability of crop yields (Jiao et al., 2019; Hassani et al., 2021; Li et al., 2021). As crucial regulatory proteins in signaling networks involved in adaptation to various abiotic pressures, 14-3-3 proteins have recently attracted considerable interest. In the current study, a 14-3-3 gene (*MdGRF6*) that negatively regulated salt stress tolerance in apple was identified (**Figures 6**, **7**).

The 14-3-3 proteins contained highly conserved proteins that are widely expressed in all eukaryotes. The family has fifteen and eight members in the *Arabidopsis* and rice genomes, respectively (DeLille et al., 2001; Rosenquist et al., 2001; Yao et al., 2007). Previously, eighteen or twenty *Md14-3-3* gene family members have been identified in the apple genome (Ren et al., 2019; Zuo et al., 2021). Here, twenty *Md14-3-3* gene*s* were identified in the apple (**Table S1**), similar to Ren et al. (2019). However, the *MD17G1105100* could not be cloned due to either its low expression level or due its non-existence. The variations in the raw high-throughput genomic sequences were likely due to splicing errors of the DNA fragments. Additionally, variations in apple germplasm resources cannot be completely ruled out (Herndon et al., 2020; Kuo et al., 2020). Previous studies have shown that 14-3-3 proteins have a significant impact on stress resistance (Roberts, 2003). In rice, OsGF14b improves salt tolerance by interacting with OsPCL1, inhibiting its ubiquitination for protection from degradation, and promoting its activity and stability (Wang et al., 2023). Overexpression of both heterologous *PvGF14a* and *PvGF14g* in transgenic *Arabidopsis* under salt stress reduces seed germination and fresh seedling weight, suggesting the involvement of these genes in the negative regulation of salt tolerance in seedlings (Li et al., 2018). In *Arabidopsis thaliana*, *GRF3* is crucial in osmotic stress response and root growth, as well as negatively regulating mitochondrial retrograde control of *AOX1a* expression *via* the ROS pathway (Li et al., 2022). Notably, we observed that *MdGRF6* was homologous to *AtGRF3* and both belonged to the non-ϵ group (**Figure 1A**). In the present study, *MdGRF6* is a negative regulator and its expression was reduced due to salt stress, similar to *AtGRF3*. The 14-3-3 protein family, therefore, exhibits high conservation of orthologs among different species (Zhou et al., 2014; Li et al., 2018).

Salt stress induces cell membrane damage primarily through osmotic and ion stresses (Golldack et al., 2014; Yoshida et al., 2014). Multiple evidences indicate that the 14-3-3 proteins are primarily localized at the plasma membrane to modify multiple ion channels and alleviate osmotic stress (Yang et al., 2019). Using the 14-3-3 omega (At1g78300) from *Arabidopsis* as bait, ion transport proteins (Ca^2+^, K^+^, and Cl^−^) were discovered using proteomic analysis of tandem affinity purified 14-3-3 protein complexes (Chang et al., 2009). Relative electrical conductivity reflects the state of the plant membrane system. An increase in the conductivity of the external medium suggests cell membrane damage and ion outflow (Ilik et al., 2018). Similarly, MDA content is an essential indicator of lipid peroxidation in plants and reflects their resistance to external adversity. When various enzymes and membrane systems in plant tissues are disrupted, MDA content increases (Hernandez et al., 2010; Mo et al., 2016). In the present study, the MDA content and relative electrical conductivity were higher in *MdGRF6-OE* calli and tobacco tissues compared to WT under salt stress. Thus, this study further confirmed that overexpression of *MdGRF6* accelerated membrane damage at high salinity.

Under abiotic stresses, plants commonly produce a large amount of ROS, which can damage the mitochondria, chloroplasts, and cell membranes unless the ROS are promptly removed (Li et al., 2015; Jia et al., 2019). Protective substances, such as SOD and POD, can scavenge ROS to alleviate salt stress and protect plant cell membrane structure (Liang et al., 2017; Guo et al., 2020). Many studies have shown that 14-3-3 proteins play a significant role in protection from osmotic and oxidative stress in plants (Yan et al., 2004). For example, RBOHD-dependent H_2_O_2_ operates upstream of H^+^-ATPase and 14-3-3 proteins, required for salt tolerance in pumpkin (Huang et al., 2019). Here, we observed that the activities of antioxidant enzymes SOD and POD were inhibited in the *MdGRF6-OE* transgenic tobacco (**Figures 6F, G**). In addition, ROS-induced cellular damage leads to the destruction of photosynthetic machinery including chlorophyll and carotenoids (Cardenas-Perez et al., 2020). Just like this, the chlorophyll contents were markedly decreased in the transgenic tobacco lines compared to WT (**Figures 5F**, **6D**). In *Arabidopsis*, it was found that 14-3-3 proteins can respond to salt stress by regulating the activity of antioxidant enzymes (Visconti et al., 2019; Wang et al., 2019). In general, 14-3-3 proteins can interact with many TFs to regulate salt stress resistance in plants (Chang et al., 2019; Wang et al., 2023). For example, 14-3-3 proteins can interact with GmMYB173 to regulate antioxidant enzyme activity and hydrogen peroxide scavenging in soybean to regulate salt resistance (Pi et al., 2018). Just like this, MdGRF6 protein may regulate the activity of antioxidant enzymes in response to salt stress by interacting with these TFs. In addition, many studies have also confirmed that 14-3-3 proteins can regulate the expression of antioxidant enzyme gene in response to stresses (Manosalva et al., 2011; Li et al., 2014). In apple, many genes such as *MdMYB46*, *MdWRKY30*, and *MdHB*-*7* are important components of the gene network that is involved in the regulation of reactive oxygen species-related genes, and they regulate ROS scavenging in response to salt stress (Chen K. et al., 2019; Dong et al., 2020; Zhao et al., 2021). Here, we found that the expression of *MdMYB46*, *MdWRKY30*, and *MdHB-7* was decreased in *MdGRF6-OE* plants, and increased in *MdGRF6-RNAi* plants (**Figures 8F–H**). This may be due to the fact that MdGRF6 interacts with other proteins to indirectly regulate their expression to modulate salt stress resistance.

Previous studies have shown that many genes can also affect ionic balance (Na^+^/K^+^ ratio) pathways in response to salt stress (Chen Z. X. et al., 2019). For example, the *MdSOS2*, *MdSOS3*, *MdNHX1*, *MdATK2/3*, and *MdCBL-1* were reported to involve in salt stress response (Shabala, 2013; Hu et al., 2016; Borkiewicz et al., 2020; Su et al., 2020). We found that the *MdSOS2*, *MdSOS3*, *MdNHX1*, *MdATK2/3*, and *MdCBL-1* genes were upregulated in *MdGRF6-RNAi* transgenic calli, but decreased in *MdGRF6-OE* lines under salt stress conditions (**Figures 8A–E**). Many functional studies have demonstrated that 14-3-3 proteins interact with the WRKY family (Chang et al., 2009; Rushton et al., 2010). In *Fortunella crassifolia*, FcWRKY40 plays an active role in salt tolerance by directly regulating *SOS2* and *P5CS1* to control ion homeostasis (Dai et al., 2018). 14-3-3 interacts with the SOS pathway proteins to cope with high salinity, attenuating interactions with AtSOS2 and activating AtSOS2 kinase activity under salt stress conditions in *Arabidopsis* (Yang et al., 2019). Therefore, we speculated that *MdGRF6* may directly regulate the expression of those genes, or indirectly regulate them by interacting with WRKY-TFs.

In this study, the mechanism by which *MdGRF6* elevates the salt sensitivity of apple possibly by regulating the activity of the antioxidant enzymes and expression of salt stress-responsive genes were investigated. In conclusion, the molecular biological functions of *MdGRF6* were investigated to provide a basis for a more in-depth exploration of the molecular mechanisms of salt stress in apples. Moreover, this study enhanced the understanding of the biological functions of the 14-3-3 gene family in apples.

## Data availability statement

The original contributions presented in the study are included in the article/**Supplementary Material**. Further inquiries can be directed to the corresponding authors.

## Author contributions

QZ and YW conceived and designed the research. YZ provided experimental materials. YZ, WK, JL, XW, LQ, and XK performed the experiments. YZ and QZ analyzed the data and wrote the manuscript. All authors contributed to the article and approved the submitted version.
