# Supplementary material for: The apple 14-3-3 gene MdGRF6 negatively regulates salt tolerance
Source: Front Plant Sci. 2023 Apr 3;14:1161539. doi: 10.3389/fpls.2023.1161539 (PMC10106762; doi:10.3389/fpls.2023.1161539)
Supplement: Supplementary file 1 [file DataSheet_1.docx]

**The apple 14-3-3 gene *MdGRF6* negatively regulates salt tolerance**

Yuqing Zhu, Wei Kuang, Jun Leng, Xue Wang, Linlin Qiu, Xiangyue Kong, Yongzhang Wang*, Qiang Zhao*

College of Horticulture, Qingdao Agricultural University, Qingdao, Shandong 266109, China; Engineering Laboratory of Genetic Improvement of Horticultural Crops of Shandong Province, Qingdao Agricultural University, Qingdao, Shandong 266109, China; Laboratory of Quality & Safety Risk Assessment for Fruit (Qingdao), Ministry of Agriculture and Rural Affairs, Qingdao Agricultural University, Qingdao, Shandong 266109, China; Qingdao Key Laboratory of Modern Agriculture Quality and Safety Engineering, Qingdao Agricultural University, Qingdao, Shandong 266109, China;

***Corresponding authors:**

1. Qiang Zhao

Address: College of Horticulture, Qingdao Agricultural University, Qingdao, Shandong 266109, China

E-mail address: [zhaoqiang000666@163.com](mailto:zhaoqiang000666@163.com)

2. Yongzhang Wang

Address: College of Horticulture, Qingdao Agricultural University, Qingdao, Shandong 266109, China

E-mail address: [qauwyz@163.com](mailto:qauwyz@163.com)

**Table S1.** The detailed information of *MdGRFs*.


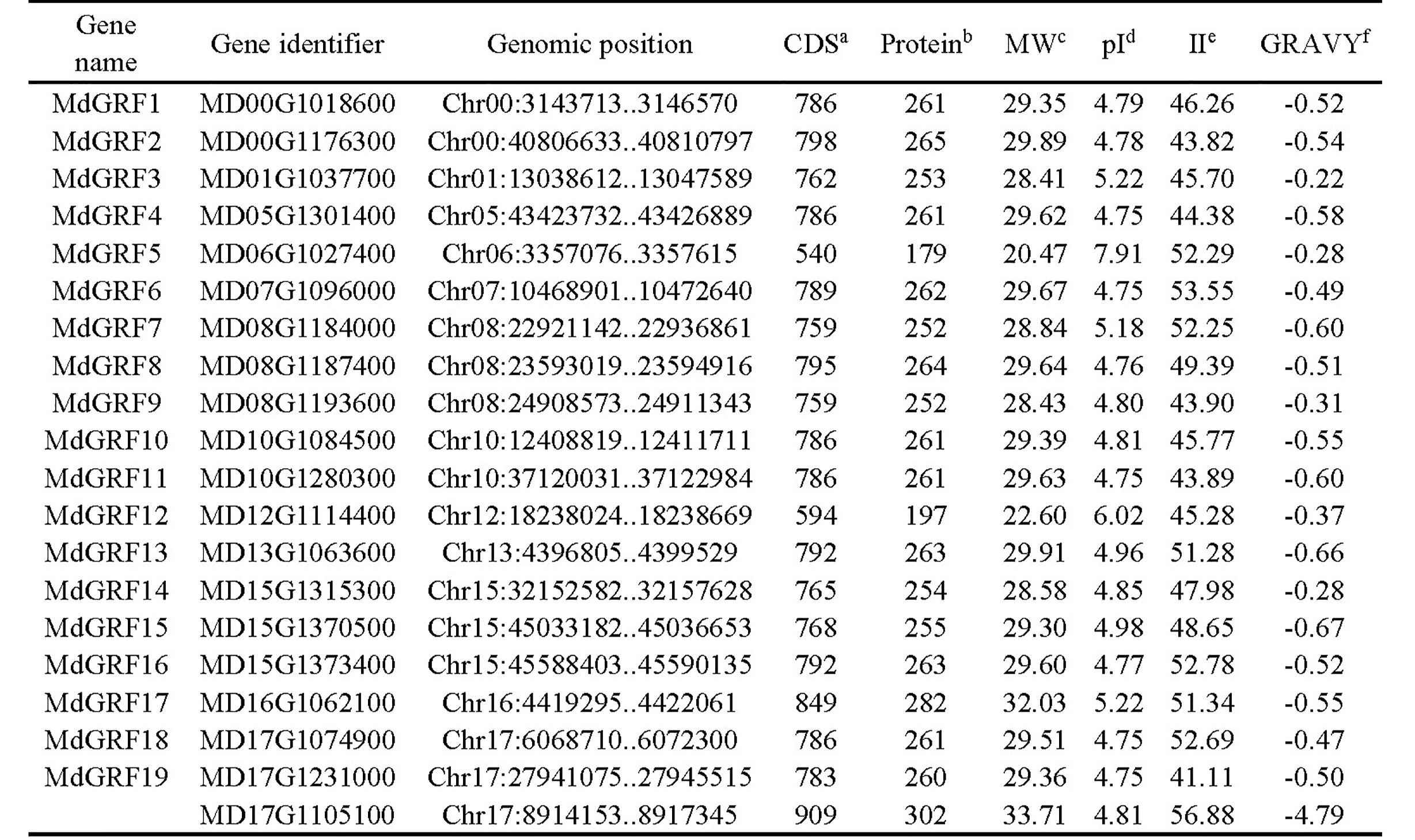


CDS^a^ (Length of the coding sequence, bp); Protein^b^ (Amino acid length, aa); MW^c^ (Molecular weight, kD); pI^d^ (Theoretical isoelectric point); II^e^ (Instability index); GRAVY^f^ (Grand average of hydropathicity).

**Tables S2.** The primers information in this study.

| Gene | Primers (5’-3’) | Notes |
| --- | --- | --- |
| *MdActin*-F | CTTCAATGTGCCTGCCATGTAT | RT-qPCR |
| *MdAction*-R | TGCTGTGCTTCACTGGATTC | RT-qPCR |
| *MdGRF1*-F | AATCCAAGCTTGTTCCTGCG | RT-qPCR |
| *MdGRF1*-R | ATTGAGGGTGTTCTCAGCGG | RT-qPCR |
| *MdGRF2-F* | CTGAACGGGAGAACTTCGCT | RT-qPCR |
| *MdGRF2-R* | GCAAGTTTCTCTCTTCAACCGT | RT-qPCR |
| *MdGRF3*-F | AGGCTGCTCAGGATATTGCG | RT-qPCR |
| *MdGRF3*-R | AAGCTTTCTCCGAGGAGTTGAG | RT-qPCR |
| *MdGRF4*-F | GAGCGAGGAATCGTACAAGGAT | RT-qPCR |
| *MdGRF4*-R | TTTAGCAGTGCCATCCACCT | RT-qPCR |
| *MdGRF5*-F | GGCTTCCGTTGTCCCTGAAA | RT-qPCR |
| *MdGRF5*-R | GATCCCGACGGTGATGGAGT | RT-qPCR |
| *MdGRF6-F* | TTGACACATTGGGTGAGGAA | RT-qPCR |
| *MdGRF6-R* | TGCTGTGCTTCACTGGATTC | RT-qPCR |
| *MdGRF7*-F | AAGAGGCCAAGGGTGGTGAA | RT-qPCR |
| *MdGRF7*-R | GGACGAAGAGGGGAGAAGGT | RT-qPCR |
| *MdGRF8*-F | GCAGAGTTGGACACTCTCGG | RT-qPCR |
| *MdGRF8*-R | GCTGCTGCTTCTTTAACCTCG | RT-qPCR |
| *MdGRF9*-F | GATGAAGGGCGACTACCACC | RT-qPCR |
| *MdGRF9*-R | AGTTGGTGACAGATCCGCAA | RT-qPCR |
| *MdGRF10*-F | ATCGAAGCTCGTTCCGTCTG | RT-qPCR |
| *MdGRF10*-R | TTCTCAGCCGCCTCTTTACG | RT-qPCR |
| *MdGRF11-F* | AACTCTCCGGAAAGGGCATGT | RT-qPCR |
| *MdGRF11-R* | AAGGTTGTCCCTCAGGAGC | RT-qPCR |
| *MdGRF12*-F | CAAGCAGAGCGCTATGAGGA | RT-qPCR |
| *MdGRF12*-R | GCCCTCCTCCTTGTTCTTGTA | RT-qPCR |
| *MdGRF13*-F | GCACCTGATTCCCTCTTCCAA | RT-qPCR |
| *MdGRF13*-R | GATGCAGCCTCGTATCCCTT | RT-qPCR |
| *MdGRF14*-F | GATTACCATCGGTACCTCGCC | RT-qPCR |
| *MdGRF14*-R | GCCAAATCAGCCAGCGCAAT | RT-qPCR |
| *MdGRF15*-F | CTTGACAGCCTCGACGAAGA | RT-qPCR |
| *MdGRF15*-R | TCTCCCTTACCTCTGGATGG | RT-qPCR |
| *MdGRF16*-F | CAGAGTTGGACACTCTCGGA | RT-qPCR |
| *MdGRF16*-R | TTGGGCGCTGCTTCTTTAAT | RT-qPCR |
| *MdGRF17*-F | CACCTGATCCCCTCTTCCTCC | RT-qPCR |
| *MdGRF17*-R | CTGCTCAGCTGCCTCTTTTCT | RT-qPCR |
| *MdGRF18*-F | GAGTTTAAGACTGGAGGCGAGA | RT-qPCR |
| *MdGRF18*-R | CCTTATTGGGTGGGTTGGAGC | RT-qPCR |
| *MdGRF19*-F | TTGCCACCTTGCAAAGACAG | RT-qPCR |
| *MdGRF19*-R | GGTCAGAAGTCCACAACGTGA | RT-qPCR |
| *MdGRF6*-F(SalI) | ttgatacatatgcccgtcgacATGTCGCCAACTGATTCTTCACG | pRI101-EGFP |
| *MdGRF6*-R(BamHI) | gcccttgctcaccatggatccCTGCTGTGCTTCACTGGATTCG | pRI101-EGFP |
| *MdGRF6*-FF(XbaI) | gagaacacgggggactctagaTATTGCCCTGGCTGAGCT | pRNAi |
| *MdGRF6*-FR(SalI) | tggggttcccccggggtcgacTTACTGCTGTGCTTCACT | pRNAi |
| *MdGRF6*-RF(KpnI) | gggttcgaaatcgatggtaccTTACTGCTGTGCTTCACT | pRNAi |
| *MdGRF6*-RR(SacI) | cgatcggggaaattcgagctcTATTGCCCTGGCTGAGCT | pRNAi |
| *Pro_MdGRF6_*-F(SalI) | caagcttggctgcaggtcgacAAAACAGCGAACGAAAGCGT | pCambia1391 |
| *Pro_MdGRF6_*-R(BamHI) | tcttagaattcccggggatccGCCGAAGCAAACGCGATAC | pCambia1391 |
| *NtActin*-F | AGGGATGCGAGGATGGA | RT-qPCR |
| *NtActin*-R | CAAGGAAATCACCGCTTTGG | RT-qPCR |
| *MdSOS2*-F | CAAAAGCACCATTCTCAAGCAC | RT-qPCR |
| *MdSOS2*-R | CCGACCAGCCAAAACCTCT | RT-qPCR |
| *MdSOS3*-F | AAGGCAAGGCGGCAGTTT | RT-qPCR |
| *MdSOS3*-R | GCGAGGCATTGGGATGAA | RT-qPCR |
| *MdNHX1*-F | AAGCGACAGTCCTGGAACATCAGT | RT-qPCR |
| *MdNHX1*-R | TATTATCACTTGCTGCCGGAGGCT | RT-qPCR |
| *MdATK2/3*-F | TTCAAGGGAAACACTTCTGC | RT-qPCR |
| *MdATK2/3*-R | TCTCTCTCCATCTCACAATCAA | RT-qPCR |
| *MdWRKY30*-F | AAGGGAGGGATCTGGCTAGG | RT-qPCR |
| *MdWRKY30*-R | TGAGGCTGAGCTGCTAGAGT | RT-qPCR |
| *MdCBL1*-F | GAGGGTGAGCTGAACGTTTG | RT-qPCR |
| *MdCBL1*-R | CGAAGCAAGACTCACGGGAT | RT-qPCR |
| *MYB46*-F | GGTTCATTACTCCCGGGCAA | RT-qPCR |
| *MYB46*-R | GGTTAGGCCGCCATGATTT | RT-qPCR |
| *MdHB7*-F | TGAGCAATGGCATTGACGGTGAG | RT-qPCR |
| *MdHB7*-R | GCAGACTCCACTAAGTTCGCAAGG | RT-qPCR |


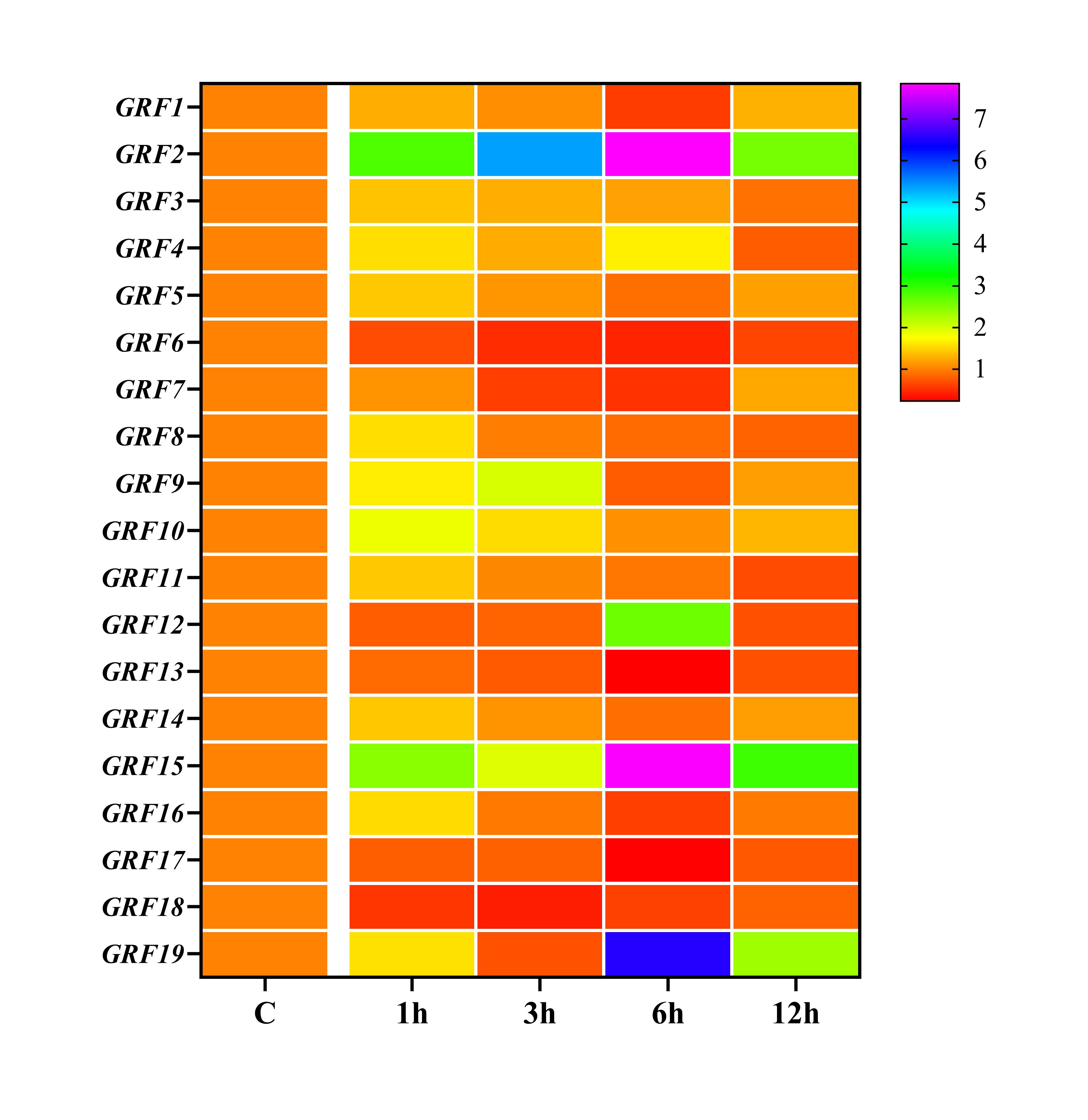


**Figure S1.** Transcriptome analysis of *Md14-3-3* genes due to salt stress. The heatmap was generated by GraphPad prism 8 and the color gradient indicates the gene expression level.


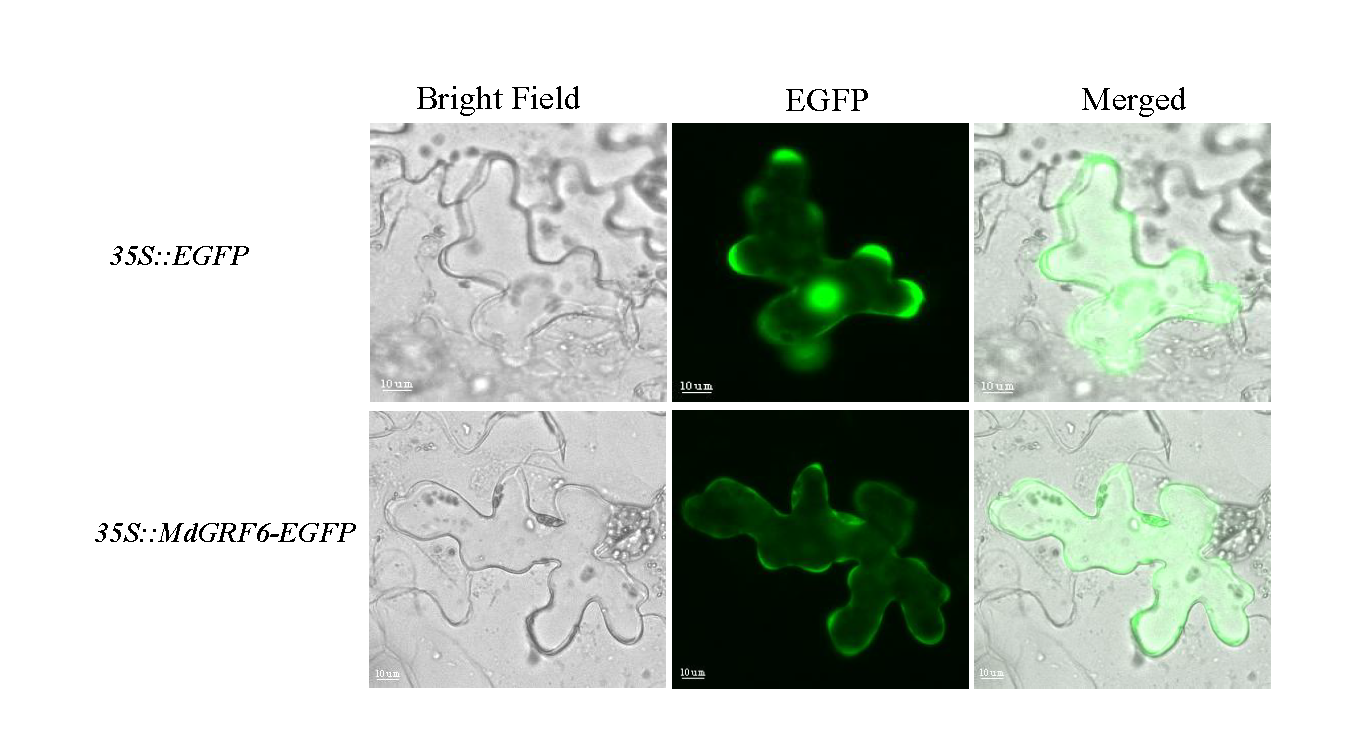


**Figure S2.** Subcellular localization of *MdGRF6* protein. The *35S::MdGRF6-EGFP* or *35S::EGFP* alone fusion constructs were expressed in tobacco epidermal cells and visualized using a confocal fluorescence microscope. Scale bar, 10 μm.


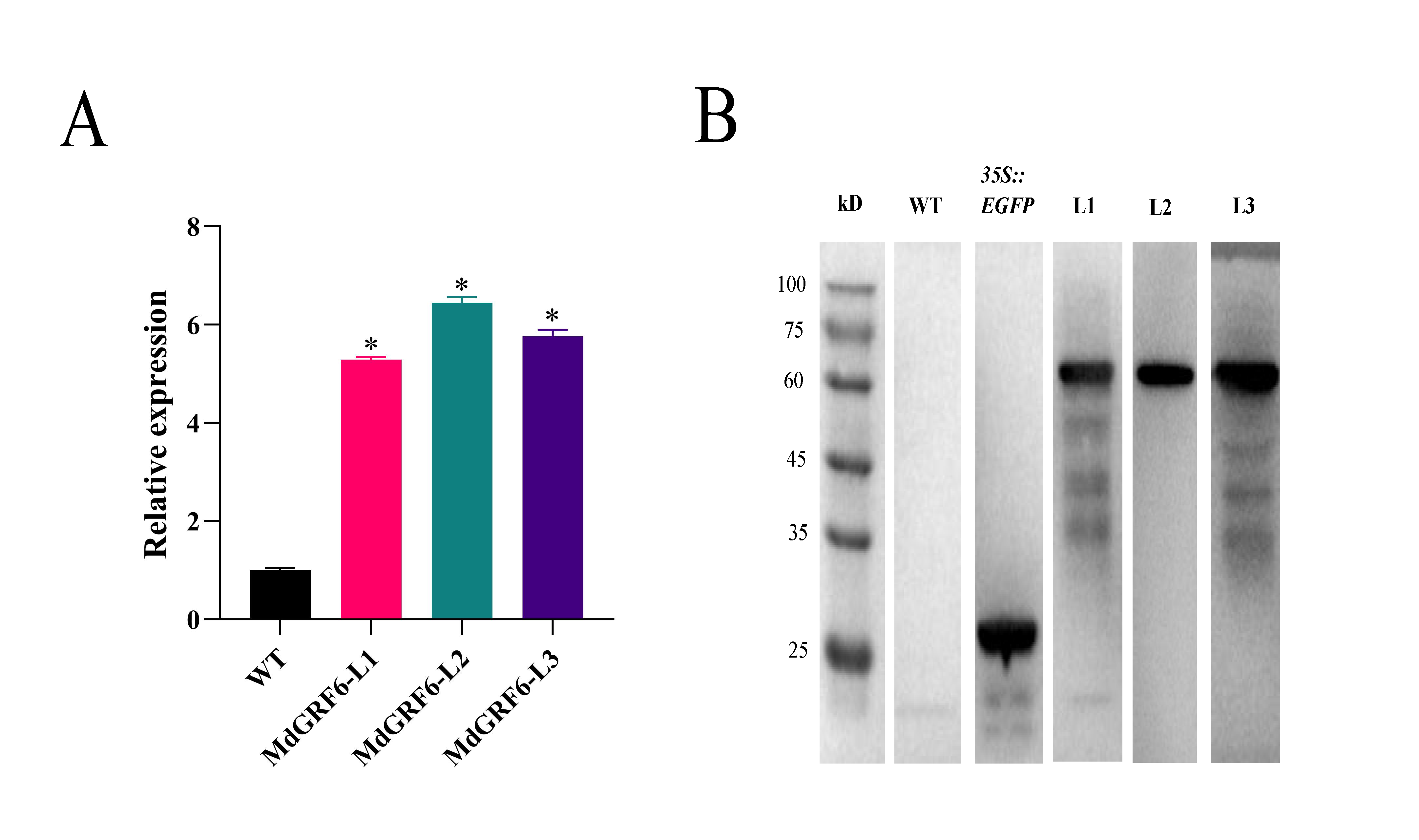


**Figure S3.** RT-qPCR and Western blotting analysis of *MdGRF6* in transgenic tobacco. (A) RT-qPCR analysis of *MdGRF6* expression in WT and *35S::MdGRF6* transgenic tobacco plants. The results were normalized to the *NtActin.* Error bars indicate the means ± SD (n = 3). The asterisks indicate significant differences (LSD test, *, P < 0.05).

(B) Total proteins from WT, *35S::EGFP* and *35S::MdGRF6-EGFP* transgenic tobacco were extracted for Western blotting.


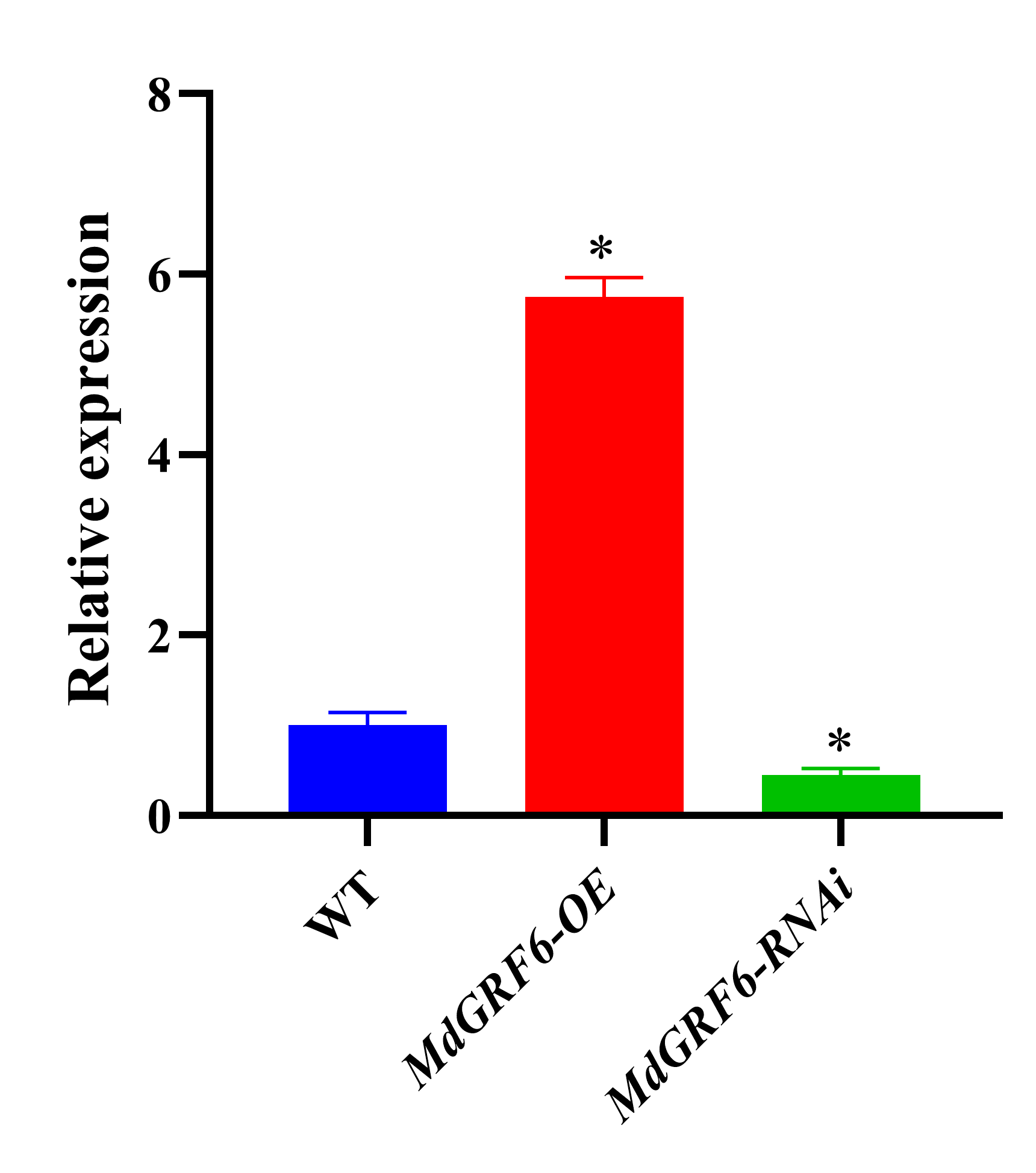


**Figure S4.** Analysis of *MdGRF6* expression in WT and transgenic calli. Error bars indicate the means ± SD (n = 3). The asterisks indicate significant differences (LSD test, *, P < 0.05).
